# Advantages of prophylactic versus conventionally scheduled heart failure therapy in an experimental model of doxorubicin-induced cardiomyopathy

**DOI:** 10.1186/s12967-019-1978-0

**Published:** 2019-07-19

**Authors:** Mária Lódi, Dániel Priksz, Gábor Áron Fülöp, Beáta Bódi, Alexandra Gyöngyösi, Lilla Nagy, Árpád Kovács, Attila Béla Kertész, Judit Kocsis, István Édes, Zoltán Csanádi, István Czuriga, Zoltán Kisvárday, Béla Juhász, István Lekli, Péter Bai, Attila Tóth, Zoltán Papp, Dániel Czuriga

**Affiliations:** 1 Division of Clinical Physiology, Department of Cardiology, Faculty of Medicine, University of Debrecen, Debrecen, Hungary; 2 Department of Pharmacology and Pharmacotherapy, Faculty of Medicine, University of Debrecen, Debrecen, Hungary; 3 Department of Pharmacology, Faculty of Pharmacy, University of Debrecen, Debrecen, Hungary; 4 Department of 3rd Internal Medicine, Semmelweis University, Budapest, Hungary; 5 Oncoradiology Center, Bács-Kiskun County Hospital, Kecskemét, Hungary; 6 Division of Cardiology, Department of Cardiology, Faculty of Medicine, University of Debrecen, Móricz Zs. krt. 22, H-4032 Debrecen, Hungary; 7 Department of Anatomy, Histology and Embryology, Faculty of Medicine, University of Debrecen, Debrecen, Hungary; 8 MTA-DE Lendület Laboratory of Cellular Metabolism, Debrecen, Hungary

**Keywords:** Animal model, Apoptosis, Cardio-oncology, Cardioprotection, Cardiotoxicity, Doxorubicin

## Abstract

**Background:**

Chemotherapy-induced left ventricular dysfunction represents a major clinical problem, which is often only recognised at an advanced stage, when supportive therapy is ineffective. Although an early heart failure treatment could positively influence the health status and clinical outcome, there is still no evidence of routine prophylactic cardioprotection for the majority of patients without previous cardiovascular history awaiting potentially cardiotoxic chemotherapy. In this study, we set out to investigate whether a prophylactic cardioprotective therapy relative to a conventionally scheduled heart failure treatment is more effective in preventing cardiotoxicity in a rodent model of doxorubicin (DOX)-induced cardiomyopathy.

**Methods:**

Male Wistar rats (*n *= 7–11 per group) were divided into 4 subgroups, namely negative controls receiving intravenous saline (CON), positive controls receiving intravenous DOX (6 cycles; D-CON), and DOX-treated animals receiving either prophylactic (PRE, started 1 week before DOX) or conventionally applied (POST, started 1 month after DOX) combined heart failure therapy of oral bisoprolol, perindopril and eplerenone. Blood pressure, heart rate, body weight and echocardiographic parameters were monitored in vivo, whereas myocardial fibrosis, capillarisation, ultrastructure, myofilament function, apoptosis, oxidative stress and mitochondrial biogenesis were studied in vitro.

**Results:**

The survival rate in the PRE group was significantly improved compared to D-CON (p = 0.0207). DOX increased the heart rate of the animals (p = 0.0193), while the blood pressure (p ≤ 0.0105) and heart rate (p = 0.0029) were significantly reduced in the PRE group compared to D-CON and POST. The ejection fraction remained preserved in the PRE group compared to D-CON or POST (p ≤ 0.0237), while none of the treatments could prevent the DOX-induced increase in the isovolumetric relaxation time. DOX decreased the rate of the actin-myosin cross-bridge cycle, irrespective of any treatment applied (p ≤ 0.0433). The myocardium of the D-CON and POST animals displayed pronounced ultrastructural damage, which was not apparent in the PRE group (p ≤ 0.033). While the DOX-induced apoptotic activity could be reduced in both the PRE and POST groups (p ≤ 0.0433), no treatment was able to prevent fibrotic remodelling or the disturbed mitochondrial biogenesis.

**Conclusion:**

For attenuating DOX-induced adverse myocardial effects, prophylactic cardioprotection has many advantages compared to a late-applied treatment.

**Electronic supplementary material:**

The online version of this article (10.1186/s12967-019-1978-0) contains supplementary material, which is available to authorized users.

## Background

Worldwide, cancer is the second leading cause of death after cardiovascular diseases, but in 12 European Union countries it has recently taken the lead [1]. Although the survival rate of patients suffering from oncological diseases has significantly risen due to developments in modern oncotherapy, the cardiovascular side effects of chemotherapy and radiotherapy have placed limits on the success of oncotherapeutic strategies. Despite intensive ongoing research efforts, chemotherapy-induced cardiotoxicity remains an unresolved clinical problem, which may lead to cardiomyopathy and consequently, heart failure, with an incidence varying over a wide range (0.2–48%) [2]. In recent years, the use of classic cytotoxic agents such as anthracyclines has been re-evaluated due to the evolving role of targeted and immuno-oncology therapies, however, they still have basic and important role in the treatment of many malignancies. Unsurprisingly, most cardio-oncology related basic research has focused on doxorubicin (DOX), which belongs to the group of anthracyclines and is one of the most commonly applied chemotherapeutic agents with well-known cardiotoxic side effects that calls for efficient therapeutic approaches to circumvent cardiotoxicity.

In tumour cells, the primary cytotoxic effect of DOX is executed through the inhibition of topoisomerase II and DNA intercalation leading to reactive oxygen species production, DNA cross-linking and apoptosis [3]. The basic mechanism underlying DOX cardiotoxicity has not yet been completely elucidated, but one of the best accepted theories is that DOX interacts with iron metabolism and this leads to the formation of an anthracycline-iron complex, which then induces lipid peroxidation, SH oxidation and DNA damage by reactive oxygen species production that in turn leads to contractile impairment, irreversible myocardial damage and fibrosis [4]. At the same time, other domains of DOX cardiotoxicity have also been proposed such as apoptosis, necrosis, inflammation, mitochondrial damage, myofilament protein dysfunction, extracellular matrix remodelling, intracellular Ca^2+^ dysregulation, etc. [5, 6].

Although many previous studies tested the hypothetical cardioprotective effects of various drug agents used in heart failure [7–16], as well as non-heart failure related substances in rodent models of DOX-induced cardiomyopathy [17–32], no systematic investigation has been conducted to evaluate the effects of a clinically relevant combination therapy and no optimum timing of preventive measures has been established. Also, numerous previous studies disregarded many clinical aspects of human cancer management, hence they did not closely mimic the human pathology (e.g. a single high dose instead of consecutive cycles of DOX, intraperitoneal instead of intravenous DOX administration, cardioprotective drug in the drinking water supply instead of oral gavage, etc.). Thus, a recent editorial stressed the need for horizontally integrating translational research for anthracycline cardiotoxicity and highlighted key criteria of experimental design, such as repetitive DOX cycles, intravenous administration, prophylactic cardioprotectant regimen, etc. [33].

As for clinical prevention, the prophylactic application of pharmacological agents with a mortality benefit in heart failure may in theory prevent or attenuate the degree of myocardial injury induced by DOX chemotherapy. Secondary prevention of cardiotoxicity has already entered clinical practice when symptoms, increase in cardiac biomarkers or overt heart failure develop, however, primary prevention is still in the research domain. One difficulty is the fact that a significant number of patients receiving chemotherapy are diagnosed with symptomatic heart failure already at an advanced stage, where the secondary preventive cardioprotective approach is incapable of reverting the adverse myocardial changes. Apparently, the prophylactic use of some studied substances (angiotensin converting enzyme inhibitors, angiotensin receptor blockers, β-blockers) have been suggested in recent position statements, but exclusively for high risk patients with multiple risk factors for cardiotoxicity, who represent only a small fraction of the oncological population [2, 34, 35]. As a routine primary prophylaxis is not yet generally recommended in the larger population mainly due to the lack of sufficient randomised data from trials with an appropriate sample size, further studies are essential to elucidate the cardioprotective effects of these drugs, their effective primary preventive dosage, the optimum timing of therapy and possible unwelcome side effects as well [36]. Although smaller human trials on preventing cardiotoxicity mostly with single heart failure agents in patients undergoing chemotherapy are already available [37–46], the results are controversial and routine prophylactic cardioprotection has not yet entered clinical practice in case of the vast majority of oncological patients without previous cardiovascular history. At present, only patients with symptoms or increased cardiac biomarker levels, or those at higher risk would qualify for treatment with heart failure medication, as recommended by current consensus guidelines in effect [2, 34, 35]. With the current study, we challenge this practice and suggest that a prophylactic cardioprotective approach preceding the commencement of a potentially cardiotoxic chemotherapy may be beneficial in the unselected larger population as well.

To mimic the human pathology and clinical management we developed a rat model of DOX-induced cardiomyopathy by applying a consecutive intravenous dosing protocol directly extrapolated from human chemotherapy. A set of in vivo and in vitro methodologies was employed to characterise myocardial changes of the animals, as well as to test the cardioprotective effects of a prophylactic heart failure treatment relative to a conventionally scheduled one commenced only at a later stage. Our primary hypothesis was that the preventive application of a combined supportive heart failure therapy preceding the cardiotoxic exposure may be more protective than that scheduled at a time when overt cardiac disease has already developed.

## Methods

### Animal experiments and study design

**Fig. 1:**
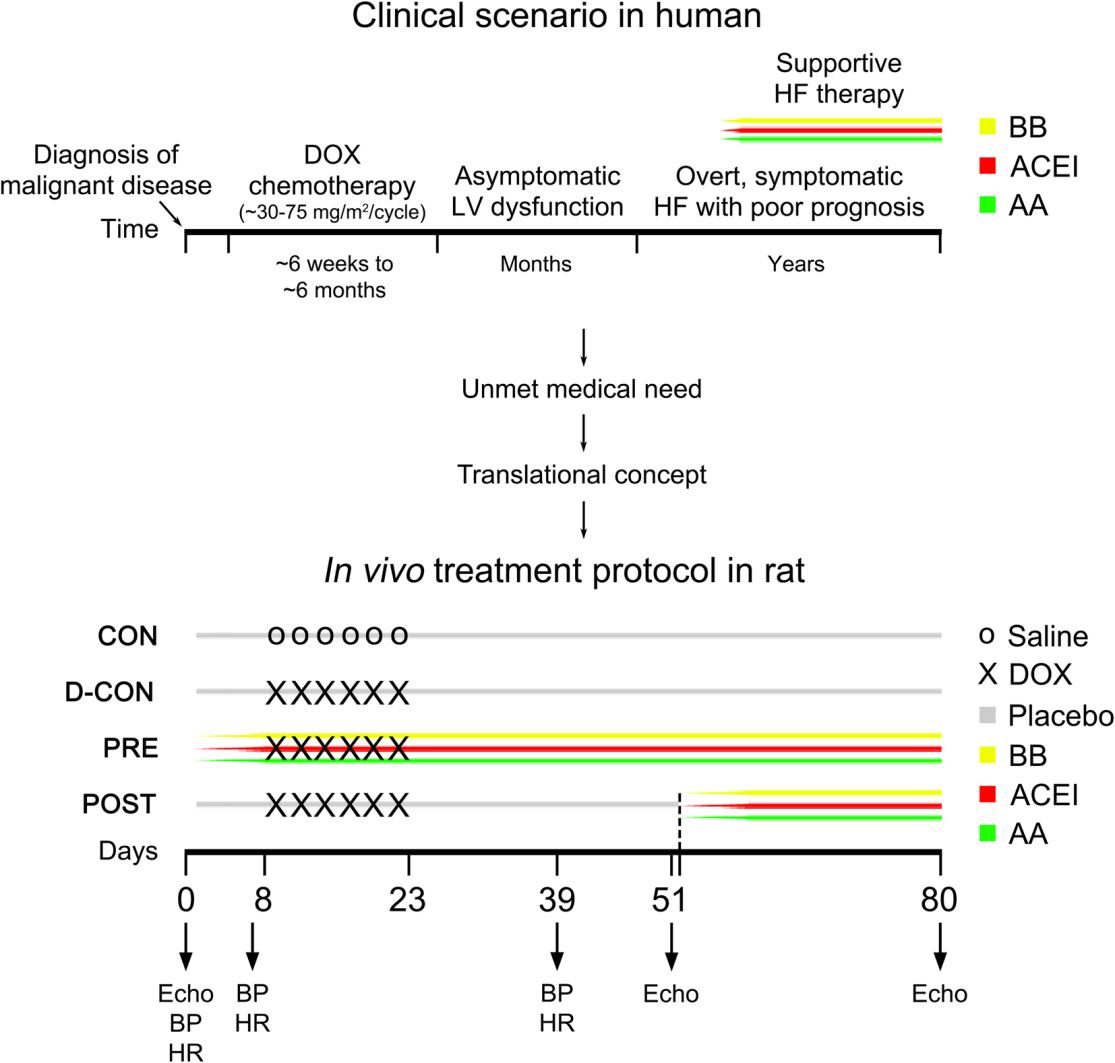
Translational concept and in vivo study protocol. On the upper timeline, cardiac deterioration, symptom development and clinical management of a hypothetical oncological patient suffering from DOX-induced cardiotoxicity can be seen, who was diagnosed with heart failure at an advanced stage resulting in a poor prognosis. On the lower timeline, the in vivo study protocol of the animals can be seen based on a translational concept. Following baseline blood pressure, heart rate measurements and echocardiography, animals in the PRE group received a daily combination of gradually uptitrated oral bisoprolol, perindopril and eplerenone started a week before DOX, while those in the POST group had the same therapy started 1 month after the intravenous DOX treatment. According to our concept, the PRE treatment represents a prophylactic cardioprotective approach for healthy subjects, which measure is currently missing from human practice, while the POST group represents subjects diagnosed with heart failure already at an advanced stage, where supportive therapy is ineffective. Animals in the CON and D-CON groups received a drug-free vehicle (“placebo”) orally throughout the study, while those in the POST group had it until day 51, then the drug-free vehicle was switched to an active heart failure therapy. The intravenous DOX exposure was carried out on days 8, 11, 14, 17, 20 and 23 in the D-CON, PRE and POST groups, while animals in the CON group received intravenous saline on the same days. Follow-up blood pressure and heart rate measurements were performed on days 7 and 39, while follow-up echocardiography was carried out on days 51 and 80. *AA* aldosterone antagonist (eplerenone), *ACEI* angiotensin converting enzyme inhibitor (perindopril), *BB* β-blocker (bisoprolol), *DOX* doxorubicin, *Echo* echocardiography, *HF* heart failure, *LV* left ventricular

In our study, efforts were made to mimic the human pathology as close as possible. Our translational concept and in vivo study protocol are presented on Fig. 1. Timing and dosing of intravenous DOX cycles were calculated from existing human chemotherapy protocols and they were corrected to the lifespan, metabolism and body surface of rats [47]. Twelve-week-old Wistar rats (*n *= 7–11 per group) were used and divided into 4 subgroups. To avoid previously described effects of hormonal differences in DOX cardiotoxicity [48], male rats were used exclusively in our study. The blood pressure (BP) and heart rate (HR) were monitored during the study by the tail-cuff method (CODA non-invasive blood pressure monitoring system, Kent Scientific Corporation, Torrington, CT, USA). Following baseline BP, HR measurements and echocardiography, animals in the prophylactic group received a daily combination of gradually uptitrated oral bisoprolol (2.5 mg/kg), perindopril (2 mg/kg) and eplerenone (6.25 mg/kg) started a week before DOX (PRE), while those in the post-exposure group had the same therapy started 1 month after the intravenous DOX treatment (POST). According to our concept, the PRE treatment represents a prophylactic cardioprotective approach for healthy subjects, which measure is currently missing from human practice, while the POST group represents subjects diagnosed with heart failure already at an advanced stage, where supportive therapy is often ineffective. The doses of the drugs applied were calculated from existing recommendations for human heart failure and they were corrected to the metabolism and body surface of rats [49]. To ensure effective serum levels of the cardioprotective medications, drugs were applied in a mucous vehicle by oral gavage every day. Negative controls in the CON group and positive controls in the D-CON group received a drug-free vehicle (“placebo”; mucilago hydroxyethylcellulosi) orally throughout the study, while animals in the POST group had it until day 51, then the drug-free vehicle was switched to an active heart failure therapy. The intravenous DOX exposure was carried out under light sedation (50 mg/kg ketamine, 5 mg/kg xylazine) by administering 1.5 mg/kg intravenous DOX into the tail veins of the animals in the D-CON, PRE and POST groups on 6 occasions (on the 8th, 11th, 14th, 17th, 20th and 23rd days of the experiment). Animals in the CON group received intravenous saline on the same days. Follow-up echocardiography was carried out under deep sedation (100 mg/kg ketamine, 10 mg/kg xylazine) on days 51 and 80, while follow-up BP and HR measurements were performed on the 7th and 39th days of the experiment. Following echocardiography on day 80, animals were anaesthetised by intraperitoneal thiopental (100 mg/kg), their hearts were excised and frozen in liquid nitrogen and stored at − 70 °C. In the D-CON group, 4 out of 11 surviving animals were pre-terminated on days 65–68 for clinical and ethical reasons to avoid imminent death and consequent loss of tissue material for the in vitro measurements. These animals were not included in the survival plot.

### Echocardiography

Echocardiography measurements were performed using a General Electric Vivid E9 ultrasound system equipped with a linear 14.1 MHz i13L probe (General Electric, Fairfield, CT, USA). For M mode based systolic parameters the parasternal long axis view was investigated. For diastolic and Doppler based systolic parameters, the 4-chamber view was examined. To evaluate strain parameters, a short cine loop was recorded from the 4-chamber view. Due to technical reasons and strict criteria of image quality, we used only two segments of the septum (basal and mid) to assess the strain parameters. All echocardiography images were obtained along with continuous electrocardiogram recording (limb leads).

### Histology

A small piece of the left ventricular (LV) free wall was embedded into Shandon™ Cryomatrix (Thermo Fischer Scientific, Waltham, MA, USA). Fifteen µm thick sections were cut using Cryotome™ Cryostat (Thermo Fischer Scientific, Waltham, MA, USA). After drying the sections, nuclei were stained with Mayer’s hemalum (VWR International, Radnor, PA, USA) for 10 min. Following 10 min of bluing, picrosirius red staining was performed using a 0.1% solution. After rinsing in isopropyl alcohol, sections were dehydrated and mounted using DPX (Sigma Aldrich, St. Louis, MO, USA). Slices were then investigated under an Olympus BX-50 microscope. Signs of fibrosis and capillary density were analysed from representative images using the ImageJ program (National Institutes of Health, Bethesda, Maryland, USA). Fibrosis was analysed on images at a magnification of 40×. The fibrotic area was expressed relative to the overall myocardial area using the colour threshold function of the ImageJ program. Capillary density was analysed in a blinded fashion on images obtained with a 40× objective, which met the following criteria: they represented cross-sectional cuts of myocardial sections, free of tissue wrinkles or staining artefacts. The relative number of capillaries and the relative capillary area were expressed by outlining all capillaries on the surface of the sections and also the overall myocardial area by freehand selections.

### TUNEL assay

To detect apoptosis, we used the terminal deoxynucleotidyl transferase (Tdt) nick end labelling test of the In Situ Cell Death Detection Kit, TMR (fluorescein-labelled cell markers) red (Roche, Mannheim, Germany). Apoptosis (DNA fragmentation) was detected by labelling the free 3′-OH termini with modified nucleotides in an enzymatic reaction. The enzyme Tdt catalyses the template-independent polymerisation of deoxyribonucleotides to the 3′-end of single- and double-stranded DNA. The steps of the sample preparation and imaging for TUNEL are described in Additional file 1. Apoptosis was quantified by the ratio of Tdt-positive nuclei/total nuclei in each section.

### Electron microscopy

Tissue processing for electron microscopy was performed using a modified version of Somogyi’s technique [50], which is described in Additional file 1. Representative pictures were taken using an Olympus Transmission Electron Microscope JEOL-1010 and iTEM software. The cardiomyocyte width was measured across the nucleus. To characterise DOX-induced ultrastructural changes, densitometry was employed on images displaying longitudinally sectioned myocardium acquired at a low magnification (3000×; 10–13 images/group) using the Image J program (National Institutes of Health, Bethesda, Maryland, USA) in a blinded fashion. Following a determination of the background density of an image (for white balance), cardiomyocytes were outlined by freehand selections, excluding all nuclei and any artefact. The area and mean density of each selection were then saved. The outlined DOX-induced ultrastructural myocardial changes (including myofibrillolysis, mitochondrial disintegration and vacuolisation) all resulted in a lower overall density of the cardiomyocytes compared to an undamaged, healthy myocardium segment. The averaged mean densities of apparently intact sarcomeres served for exposition control. The final, background and exposition corrected mean densities of myocardium selections were weight-averaged for their respective area on the image.

### Force measurements in isolated cardiomyocytes

The technique for force measurements in single, permeabilised cardiomyocyte preparations was described earlier [51–53]. Repeated activation–relaxation cycles were performed in single cardiomyocytes at 15 °C (to maintain the stability of the preparations), at a sarcomere length of 2.2 μm. Isometric force values were normalised for the maximal Ca^2+^-activated active force, and Ca^2+^–force relations were fitted to a modified Hill equation to determine the Ca^2+^-sensitivity of isometric-force production, i.e. pCa_50_. The active isometric force (F_max_), Ca^2+^-independent passive force (F_passive_) and the rate constant of force redevelopment (*k*_tr,max_) were then assessed. F_max_ and F_passive_ were normalised for the cardiomyocyte cross-sectional area, which was measured by using optically directed light.

### Oxidative status of contractile proteins

Protein carbonyl group investigations were adapted from Balogh et al. [53] using an Oxyblot Protein Oxydation Detection Kit (Merck Millipore, Burlington, MA, USA). The detailed steps of sample processing and signal detection are presented in Additional file 1.

### Phosphorylation status of contractile proteins

To investigate protein phosphorylation, ProQ™ Diamond protein gel staining (Thermo Fisher Scientific, Waltham, MA, USA) was employed. The steps of tissue preparation and signal detection are described in Additional file 1.

### Western immunoblot for mitochondrial proteins

Samples from the LV free wall were processed and labelled with the following antibodies being produced in a rabbit: anti-acetyl coenzyme A carboxylase (ACC), anti-phospho-ACC and anti-peroxisome proliferator-activated receptor-gamma coactivator 1 alpha (PGC1α) (Cell Signaling Technology, Boston, MA, USA). The detailed steps of tissue processing and imaging are given in Additional file 1.

### Western immunoblot for caspase-3

Approximately 100 mg of heart tissue was used and labelled with an antibody against caspase-3. The steps of tissue preparation and visualisation are given in Additional file 1.

### Data analysis and statistics

During the mechanical measurements, Ca^2+^-induced contractions of the preparations were recorded with a custom-built LabVIEW Data Acquisition platform. The contractile parameters of the cardiomyocytes were analysed with the LabVIEW analysing software package (Myo; National Instruments, Austin, TX, USA) and Origin 6.0 (Originlab Corporation, Northampton, MA, USA). The signal intensities of protein bands were quantified using the ImageJ (National Institutes of Health, Bethesda, Maryland, USA) and MagicPlot (MagicPlot Systems, Saint Petersburg, Russia) software packages. Laboratory variables were measured multiple times, averaged within each animal and used in an analysis as a single value characteristic of that animal (“mean of the mean”; except for body weight, body mass index, strain imaging and biochemical measurements on Fig. 8). The sample size of the experimental groups is indicated on the figure panels, while the number of measurements is stated in the figure and table legends. Between-groups comparisons for the survival outcome were based on an overall log-rank test and all pairwise variants thereof. For all other outcomes, analysis of variance or the Kruskal–Wallis test was applied for overall, and Student’s two-sample t test or Wilcoxon’s rank-sum test for pairwise comparisons, as appropriate for distribution shapes. Values are given as mean ± standard error of the mean (SEM). The criterion for statistical significance was p ≤ 0.05. In the experiment, the statistical package utilised was Stata (StataCorp LLC. 2017, Stata Statistical Software: Release 15. College Station, TX, USA).

## Results

**Fig. 2:**
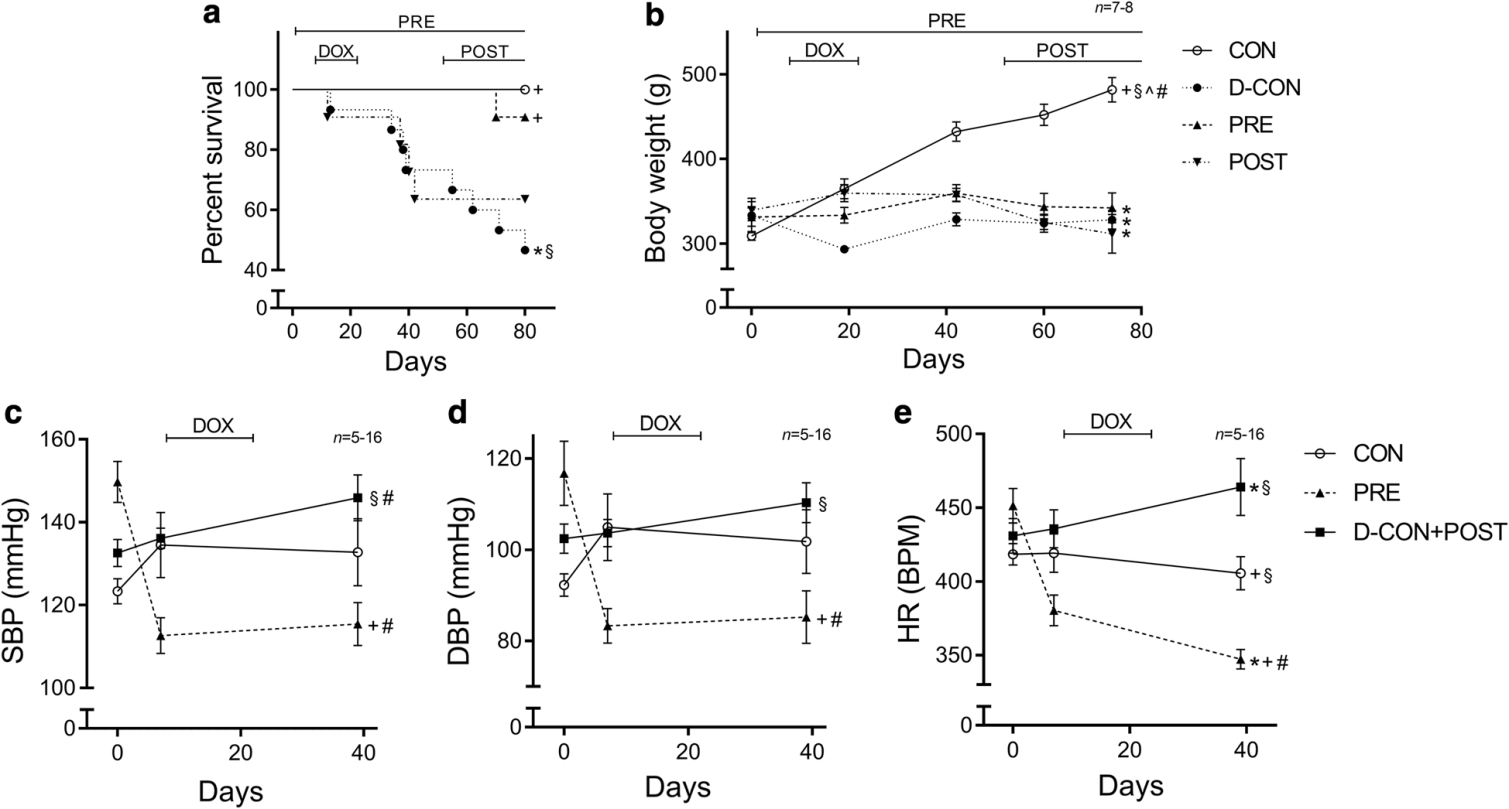
Survival and clinical parameters of the animals. The survival rate in the D-CON group was significantly worse compared to that in CON, but it remained preserved in the PRE group (**a**). The body weight was significantly lower in groups exposed to DOX compared to that in CON, irrespective of any treatment applied (**b**). DOX significantly increased the heart rate of animals, while the prophylactic treatment in the PRE group significantly decreased the blood pressure and heart rate (**c**–**e**). *DBP* diastolic blood pressure, *HR* heart rate, *SBP* systolic blood pressure. Lines at top represent doxorubicin exposure (DOX), prophylactic (PRE) or conventionally scheduled heart failure treatment (POST), respectively. *n* number of animals per group (5 measurements per animal, except for single body weight measurements); Statistics: Wilcoxon’s rank-sum test except for survival (log-rank test); *p ≤ 0.05 vs. CON; ^+^p ≤ 0.05 vs. D-CON/D-CON + POST; ^§^p ≤ 0.05 vs. PRE; ^^^p ≤ 0.05 vs. POST; ^#^p ≤ 0.05 vs. day 0

The clinical parameters of the animals are presented in detail in Table 1. The survival rate in the D-CON group was significantly worse compared to that in CON (p = 0.0247), but it remained preserved in the PRE group (p = 0.0207 vs. D-CON). Animals in the POST group were able to maintain their health status once combined heart failure therapy was commenced from the beginning of day 52 (Fig. 2a). The thriving of animals (*i.e.* putting on weight) exposed to DOX was significantly worse compared to that in CON, irrespective of any treatment applied (p ≤ 0.0018) (Fig. 2b). The heart rate and blood pressure were monitored until day 39. Afterwards the tail of the DOX-treated animals became stiff and sclerotic due to the direct effect of intravenous DOX, hindering reliable tail-cuff measurements. This is why the D-CON and POST animals were pooled during these examinations, as up until then the treatment of the animals was identical (the POST treatment began only from day 52). DOX significantly increased the heart rate of the animals compared to that in CON [464 ± 19 vs. 406 ± 11 beats per minute (BPM), p = 0.0193], while the prophylactic treatment of the PRE group significantly lowered the heart rate, the systolic and diastolic blood pressure compared to CON and/or D-CON + POST (347 ± 7 BPM, p = 0.0074 vs. CON, p = 0.0029 vs. D-CON + POST; 115 ± 5/85 ± 6 vs. 146 ± 6/110 ± 4 mmHg in PRE and D-CON + POST, respectively, p ≤ 0.0105) (Fig. 2c–e).

**Table 1:** Clinical parameters of the animals

	CON (*n *= 7)		D-CON (*n *= 7)		PRE (*n *= 8)		POST (*n *= 7)	
	Day 0	Day 74	Day 0	Day 74	Day 0	Day 74	Day 0	Day 74
*BW* (g)	309.29 ± 5.25^^^	481.71 ± 14.37^+§^#^	333.43 ± 20.42	328.29 ± 10.91*	331.38 ± 10.86	342.25 ± 49.7*	339.71 ± 10.04*	311.71 ± 22.85*
*tBMI* (kg/m^2^)	157 ± 3.57^+§^^	248.87 ± 8.74^+§^#^	194.78 ± 18.392*	189.9 ± 9.71*	187.91 ± 10.62*	189.72 ± 15.98*	206.45 ± 11.16*	190.52 ± 7.88*

	CON (*n *= 7)			D-CON + POST (*n *= 16)			PRE (*n *= 5–8)		
	Day 0	Day 7	Day 39	Day 0	Day 7	Day 39	Day 0	Day 7	Day 39
*SBP* (mmHg)	123.31 ± 3.05^§^	134.51 ± 7.85^§^	132.77 ± 8.11	132.61 ± 3.27^§^	136.11 ± 2.47^§^	145.91 ± 5.54^§#^	149.73 ± 4.98*^+^	112.63 ± 4.3*^+#^	115.44 ± 5.19^+#^
*DBP* (mmHg)	92.23 ± 2.49^§^	104.91 ± 7.35^§#^	103.34 ± 6.34	102.41 ± 3.23^§^	103.64 ± 3.01^§^	110.34 ± 4.39^§^	116.75 ± 7.06*^+^	83.3 ± 3.79*^+#^	85.24 ± 5.77^+#^
*HR* (1/min)	418.31 ± 7.27^§^	419.2 ± 13.05^§^	405.51 ± 11.18^+§^	430.86 ± 11.73	435.75 ± 12.98^§^	464.06 ± 19.36*^§^	451.25 ± 11.81*	380.33 ± 10.41*^+#^	347.24 ± 6.59*^+#^

*BW* body weight, *DBP* diastolic blood pressure, *HR* heart rate, *SBP* systolic blood pressure, *tBMI* body mass indexed for tibia length, *n* number of animals per group (5 measurements per animal, except for single body weight and tBMI measurements)

* p ≤ 0.05 vs. CON; ^+^ p ≤ 0.05 vs. D-CON/D-CON + POST; ^§^ p ≤ 0.05 vs. PRE; ^^^ p ≤ 0.05 vs. POST; ^#^ p ≤ 0.05 vs. day 0

**Fig. 3:**
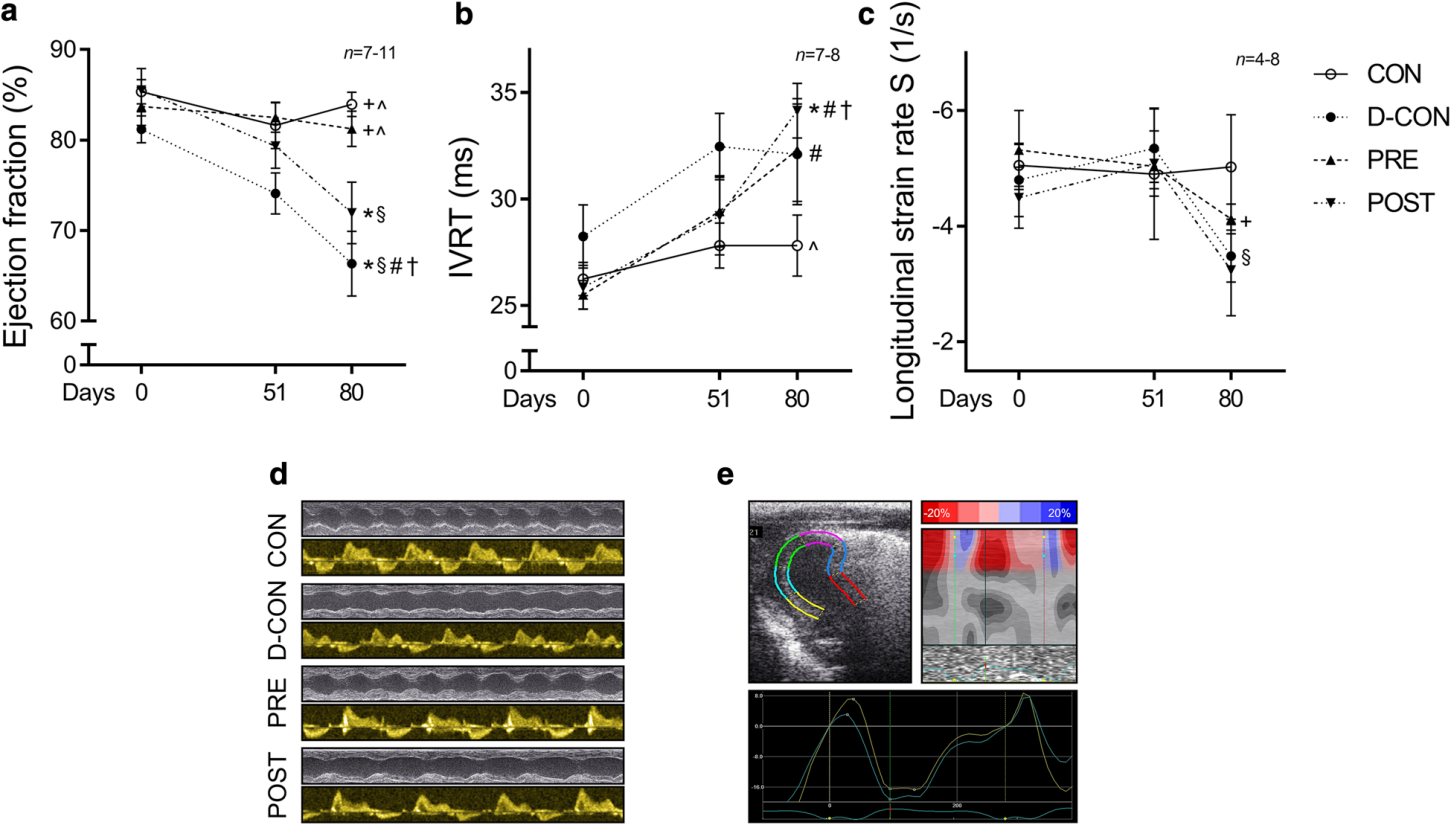
Echocardiographic parameters of the animals. At follow-up, a significantly reduced ejection fraction was seen in the D-CON and POST groups compared to CON, while the ejection fraction in the PRE group remained preserved (**a**). A slight increase in the IVRT was observed in the DOX-treated animals, which was statistically significant in the POST animals compared to that in CON (**b**). A slight decrease in the longitudinal strain rate was observed in the DOX-treated animals, which was more apparent in the D-CON and POST groups (**c**). Representative recordings of M-mode and Doppler mode echocardiography (**d**). Illustration of strain imaging in the rat heart (**e**). IVRT = isovolumetric relaxation time. *n* number of animals per group (3 measurements per animal, except for single strain measurements); Statistics: Wilcoxon’s rank-sum test; *p ≤ 0.05 vs. CON; ^+^p ≤ 0.05 vs. D-CON; ^§^p ≤ 0.05 vs. PRE; ^^^p ≤ 0.05 vs. POST; ^#^p ≤ 0.05 vs. day 0; ^†^p ≤ 0.05 vs. day 51

Echocardiographic data of the animals are presented in Table 2. At follow-up on day 80, a significantly reduced ejection fraction was seen in the D-CON group compared to that in CON (66.4 ± 3.6 vs. 84 ± 1.3%, p = 0.0043). This decrease was prevented by the prophylactic (81.3 ± 2%, p = 0.0046 vs. D-CON), but not by the conventionally scheduled treatment (72 ± 3.4%, p = 0.3886 vs. D-CON, p = 0.0237 vs. PRE) (Fig. 3a). A slight increase in the isovolumetric relaxation time (IVRT) was observed in the DOX-treated animals, which was statistically significant in the POST animals compared to that in CON (34.1 ± 1.3 vs. 27.8 ± 1.4 ms, p = 0.006) (Fig. 3b). A slight decrease in the systolic longitudinal strain rate was observed in the DOX-treated groups, which was more apparent in the D-CON and POST groups than that in PRE [− 3.5 ± 0.4, − 4.1 ± 0.3, − 3.3 ± 0.8 vs. − 5 ± 0.9 1/s in D-CON, PRE, POST and CON, respectively, p = 0.0321 (PRE vs. D-CON)] (Fig. 3c).

**Table 2:** Echocardiographic parameters of the animals

	CON (*n *= 6–7)			D-CON (*n *= 7–11)			PRE (*n *= 6–8)			POST (*n *= 4–7)		
	Day 0	Day 51	Day 80	Day 0	Day 51	Day 80	Day 0	Day 51	Day 80	Day 0	Day 51	Day 80
*EF* (%)	85.33 ± 1.34	81.62 ± 2.55^+^	83.95 ± 1.34^+^^	81.17 ± 1.50	74.1 ± 2.28*^§#^	66.35 ± 3.58*^§#†^	83.71 ± 2.11	82.5 ± 1.62^+^	81.25 ± 1.96^+^^	85.52 ± 2.4	79.33 ± 2.46	71.95 ± 3.4*^§^
*LVEDd* (mm)	5.52 ± 0.2	5.66 ± 0.23	6.39 ± 0.27^#^	5.26 ± 0.21	5.76 ± 0.3	5.89 ± 0.28^#^	5.61 ± 0.12	6.07 ± 0.19	5.88 ± 0.28	5.27 ± 0.19	5.98 ± 0.23^#^	5.89 ± 0.15
*LVESd* (mm)	2.61 ± 0.27	3.08 ± 0.22	3.34 ± 0.21^#^	2.92 ± 0.19	3.61 ± 0.28^#^	3.98 ± 0.28^#^	2.92 ± 0.15	3.27 ± 0.17	3.21 ± 0.19	2.64 ± 0.2	3.26 ± 0.22	3.73 ± 0.23^#^
*PWd* (mm)	0.86 ± 0.05	0.79 ± 0.03^+§^	0.94 ± 0.03^^†^	0.8 ± 0.02	0.91 ± 0.03*^#^	0.91 ± 0.03^^#^	0.85 ± 0.02	0.9 ± 0.02*	0.96 ± 0.05	0.87 ± 0.03	0.86 ± 0.02	1.07 ± 0.05*^+#†^
*PWs* (mm)	1.12 ± 0.05^+^	0.94 ± 0.04^§#^	1.25 ± 0.07^+†^	0.95 ± 0.03*	0.99 ± 0.03	1 ± 0.05*^^^	0.99 ± 0.04	1.09 ± 0.04*	1.14 ± 0.05^#^	1.02 ± 0.02	1.02 ± 0.03	1.28 ± 0.06^+#†^
*IVSd* (mm)	0.99 ± 0.07	0.99 ± 0.07^#^	1.44 ± 0.13^#^	1.02 ± 0.06	1.08 ± 0.09	1.28 ± 0.08^§#^	1.02 ± 0.07	1.1 ± 0.07	1.61 ± 0.13^+#†^	0.96 ± 0.04	1.02 ± 0.05	1.46 ± 0.07^#†^
*IVSs* (mm)	1.13 ± 0.05	1.25 ± 0.08	1.74 ± 0.17^+#^	1.14 ± 0.07	1.23 ± 0.09	1.33 ± 0.08*^#^	1.12 ± 0.07	1.28 ± 0.06^^^	1.72 ± 0.2^#^	1.07 ± 0.05	1.12 ± 0.03^§^	1.75 ± 0.19^#†^
*IVCT* (ms)	18.46 ± 1.29	17.81 ± 1.34	15.51 ± 0.73^+#^	18.64 ± 2.7	19.09 ± 1.59	23.66 ± 3.23*	19.46 ± 1.31	17.92 ± 1.24	16.42 ± 1.86	17.76 ± 0.75	19.43 ± 1.2	17.95 ± 0.99
*IVRT* (ms)	26.24 ± 0.78	27.81 ± 1.05^+^	27.81 ± 1.44^	28.24 ± 1.48	32.45 ± 1.56*	32.1 ± 2.37	25.5 ± 0.66	29.42 ± 1.68	32.29 ± 2.42^#^	25.86 ± 1.03	29.19 ± 1.83	34.14 ± 1.28*^#†^
*DecT* (ms)	41.79 ± 4.25	25.86 ± 4.37	50.33 ± 1.62^+^	40.33 ± 3.36	44.62 ± 2.3	42.71 ± 2.73*	41.79 ± 2.73	51 ± 1.95^#^	51.96 ± 3.96^#^	43.62 ± 2.33	43.62 ± 4.5	49.38 ± 4.81
*ET* (ms)	62.9 ± 1.58	59.62 ± 1.95^§^^	62.86 ± 1.68^+§^^	60.42 ± 1.97	68.04 ± 3.32^#^	69.46 ± 2.19*^§^#^	60.96 ± 1.35	70.25 ± 1.38*^#^	77.29 ± 2.67*^+#†^	60.48 ± 1.67	68.38 ± 2.45*^#^	78.57 ± 3.39*^+#†^
*E* (cm/s)	66.78 ± 3.14	58.33 ± 4.55	59 ± 2.83	58.79 ± 4.85	55.58 ± 3.05^§^	59.67 ± 2.41	67.25 ± 3.43	70.54 ± 4.93^+^	60 ± 3.43	65.76 ± 4.49	67.1 ± 3.7	59.71 ± 5.42
*A* (cm/s)	50.46 ± 3.16	39.57 ± 4.97	34.52 ± 2.23^^#^	42.67 ± 4.67	38.92 ± 3.14	36.71 ± 2.68^^^	50.25 ± 3.71	45.08 ± 3.48	36.63 ± 2.7^#^	47.71 ± 4.47	38.67 ± 4	26.05 ± 2.84*^+#†^
*e’* (cm/s)	4.63 ± 0.76	3.71 ± 0.29	4.43 ± 0.29^§^	3.96 ± 0.24	4.04 ± 0.3	4.33 ± 0.38	4.12 ± 0.3	3.83 ± 0.34	3.54 ± 0.26*	3.9 ± 0.24	4.29 ± 0.43	3.9 ± 0.35
*a’* (cm/s)	6.1 ± 0.48	5.57 ± 0.54^+^	3.19 ± 0.18^+§#†^	5.38 ± 0.4	4.12 ± 0.25*^#^	5.04 ± 0.84*^^^	6.08 ± 0.55	4.38 ± 0.38^#^	3.92 ± 0.22*^^#^	6.1 ± 0.61	4.67 ± 0.47	3.24 ± 0.27^+§#^
*s’* (mm/s)	30.49 ± 2.38	33.48 ± 1.94^§^^	31.33 ± 2.7^§^^	37.08 ± 5.44	33.58 ± 2.2^§^^	30 ± 1.36^§^^	31.25 ± 2.78	27.25 ± 1.4*^+^	22.42 ± 1.4*^+#†^	30 ± 2.78	25.48 ± 0.69*^+^	21.62 ± 0.83*^+#†^
*E/e’*	16.15 ± 1.96	16.13 ± 1.56	13.84 ± 1.46	15.45 ± 1.93	14.05 ± 0.86	14.56 ± 1.49	17 ± 1.67	19.68 ± 2.4	17.5 ± 1.67	17.07 ± 1.19	16.62 ± 2.02	15.98 ± 1.83
*Septal strain* (%)	–20.17 ± 4.61	–24.04 ± 1.45	–16.83 ± 2.18	–18.32 ± 2.81	–20.15 ± 1.57	–15.64 ± 1.83	–21.53 ± 2.6	–20.65 ± 1.4	–17.38 ± 2.67	–15.37 ± 4.82	–14.73 ± 3.7	–11.36 ± 3.37
*Strain rate S* (1/s)	–5.06 ± 0.36	–4.9 ± 1.12	–5.03 ± 0.9	–4.8 ± 0.63	–5.35 ± 0.69	–3.49 ± 0.44^§^	–5.32 ± 0.68	–5.04 ± 0.27	–4.13 ± 0.25^+^	–4.5 ± 0.52	–5.09 ± 0.56	–3.25 ± 0.78
*Strain rate E* (1/s)	4.82 ± 1.1	5.12 ± 0.58	4.12 ± 0.61^	5.16 ± 0.44	4.58 ± 0.72	2.33 ± 0.73^#^	6.25 ± 0.77	4.2 ± 0.53	2.87 ± 1.16	4.9 ± 0.99	4.56 ± 0.3	2.0 ± 0.3*
*Tei*-*index*	0.72 ± 0.03	0.78 ± 0.05^§^	0.69 ± 0.05	0.8 ± 0.09	0.82 ± 0.03^§^^	0.82 ± 0.06^§^^	0.73 ± 0.03	0.67 ± 0.03*^+^	0.66 ± 0.05^+^	0.71 ± 0.03	0.71 ± 0.04^+^	0.65 ± 0.03^+^

*A* mitral A wave, *a′* late diastolic velocity of the mitral anulus, *DecT* deceleration time, *E* mitral E wave, *e′* early diastolic velocity of the mitral anulus, *EF* ejection fraction, *ET* ejection time, *IVCT* isovolumetric contraction time, *IVRT* isovolumetric relaxation time, *IVSd* diastolic thickness of the interventricular septum, *IVSs* systolic thickness of the interventricular septum, *LVEDd* end-diastolic diameter of the left ventricle, *LVESd* end-systolic diameter of the left ventricle, *PWd* diastolic thickness of the posterior wall, *PWs* systolic thickness of the posterior wall, *s′* systolic velocity of the mitral anulus, *Strain rate E* strain rate measured at the time of the E wave (diastolic), *Strain rate S* strain rate measured at the time of the s*′* wave (systolic), *Tei-index* myocardial performance index [(IVCT + IVRT)/ET], *n* number of animals per group (3 measurements per animal, except for single strain measurements)

* p ≤ 0.05 vs. CON; ^+^ p ≤ 0.05 vs. D-CON; ^§^ p ≤ 0.05 vs. PRE; ^^^ p ≤ 0.05 vs. POST; ^#^ p ≤ 0.05 vs. day 0; ^†^ p ≤ 0.05 vs. day 51

**Fig. 4:**
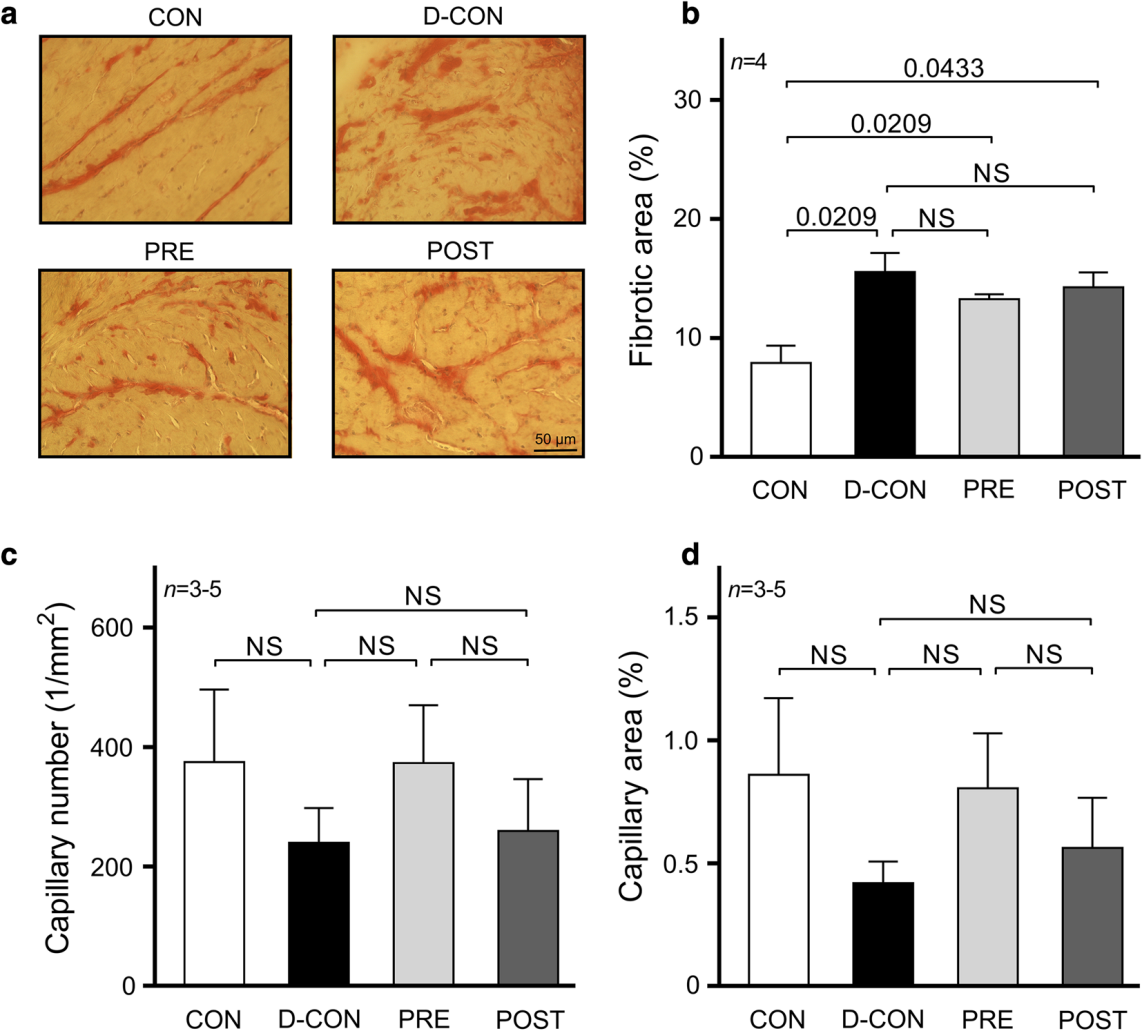
Myocardial fibrosis and capillary density. **a** Representative images of myocardial sections stained with picrosirius red and Mayer’s hemalum in all groups. The red colour identifies fibrosis. **b** Increased fibrotic area was detected in all DOX-exposed groups, irrespective of any treatment applied. **c**, **d** A mild, statistically non-significant capillary rarefaction could be detected in the D-CON and POST groups compared to CON or PRE, which was evident from the slight decrease in the relative number and relative area of capillaries. *n* number of animals per group (9 images per animal for fibrosis, 1–4 images per animal for capillary density); Statistics: Wilcoxon’s rank-sum test; numbers are p values; NS: non-significant

**Fig. 5:**
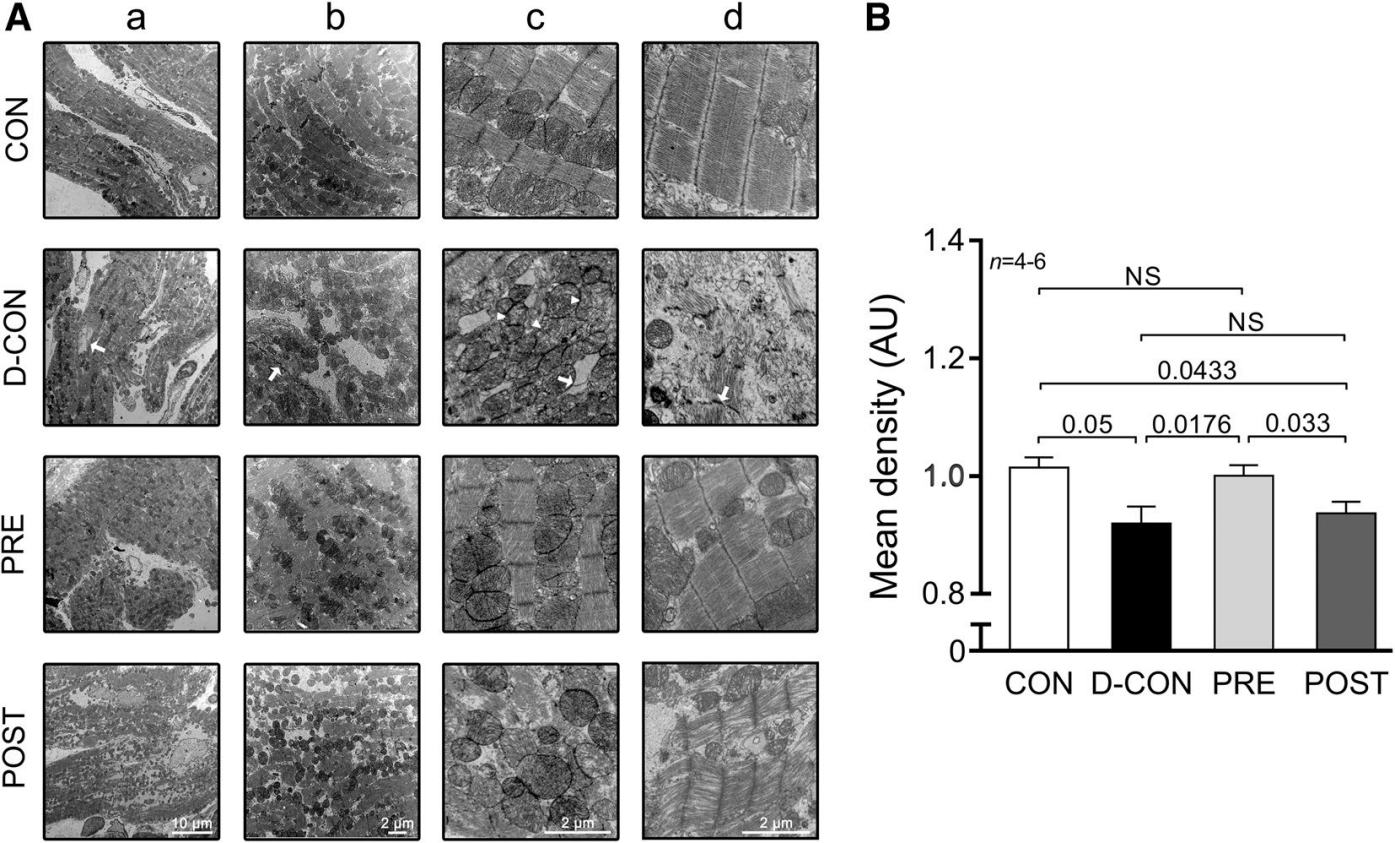
Electron microscopic imaging. **A** Pronounced ultrastructural changes were seen in the ultrathin myocardial sections obtained from the D-CON group: myofibrillolysis (d), vacuolisation (c, arrow), mitochondrial damage (c, arrowhead), Z-disc degradation (b, d, arrow) and chromatin disintegration in the nuclei (a, arrow). Samples from the POST animals displayed a visually similar ultrastructural pattern to those from the D-CON group, with less pronounced mitochondrial damage, while in the PRE group, most of the above ultrastructural changes were not apparent—except for a mild degree of myofibrillolysis. **B** Densitometry suggested a preserved myocardial ultrastructure in the PRE, but not in the POST group. *AU* optical density in arbitrary units. *n* number of animals per group (1-6 images per animal); Statistics: Wilcoxon’s rank-sum test; numbers are p values; *NS* non-significant

Picrosirius red staining revealed an increased fibrotic area (> 10%) in all DOX-exposed groups, irrespective of any treatment applied (p ≤ 0.0433) (Fig. 4a, b). A mild, statistically non-significant capillary rarefaction could be detected in the D-CON and POST groups compared to CON or PRE, which was evident from the slight decrease in the relative number and relative area of capillaries (Fig. 4c, d). On electron microscopical examination, pronounced ultrastructural changes were observed in the ultrathin myocardial sections obtained from the D-CON group, namely myofibrillolysis, vacuolisation, mitochondrial damage, Z-disc degradation and chromatin disintegration in the nuclei. Samples from the POST animals displayed a visually similar ultrastructural pattern to those of the D-CON group, with less pronounced mitochondrial damage, while in the PRE group most of the above ultrastructural changes were not apparent—except for a mild degree of myofibrillolysis (Fig. 5A). Quantification of the electron microscopic images by densitometry suggested a preserved myocardial ultrastructure in the PRE, but not in the POST group (p ≤ 0.0433) (Fig. 5B). On further analysis, a mild cardiomyocyte hypertrophy could be seen in the D-CON group (17 ± 0.9 µm), which was significant compared to that in PRE (14 ± 0.6 µm, p = 0.0301) (data not shown).

**Fig. 6:**
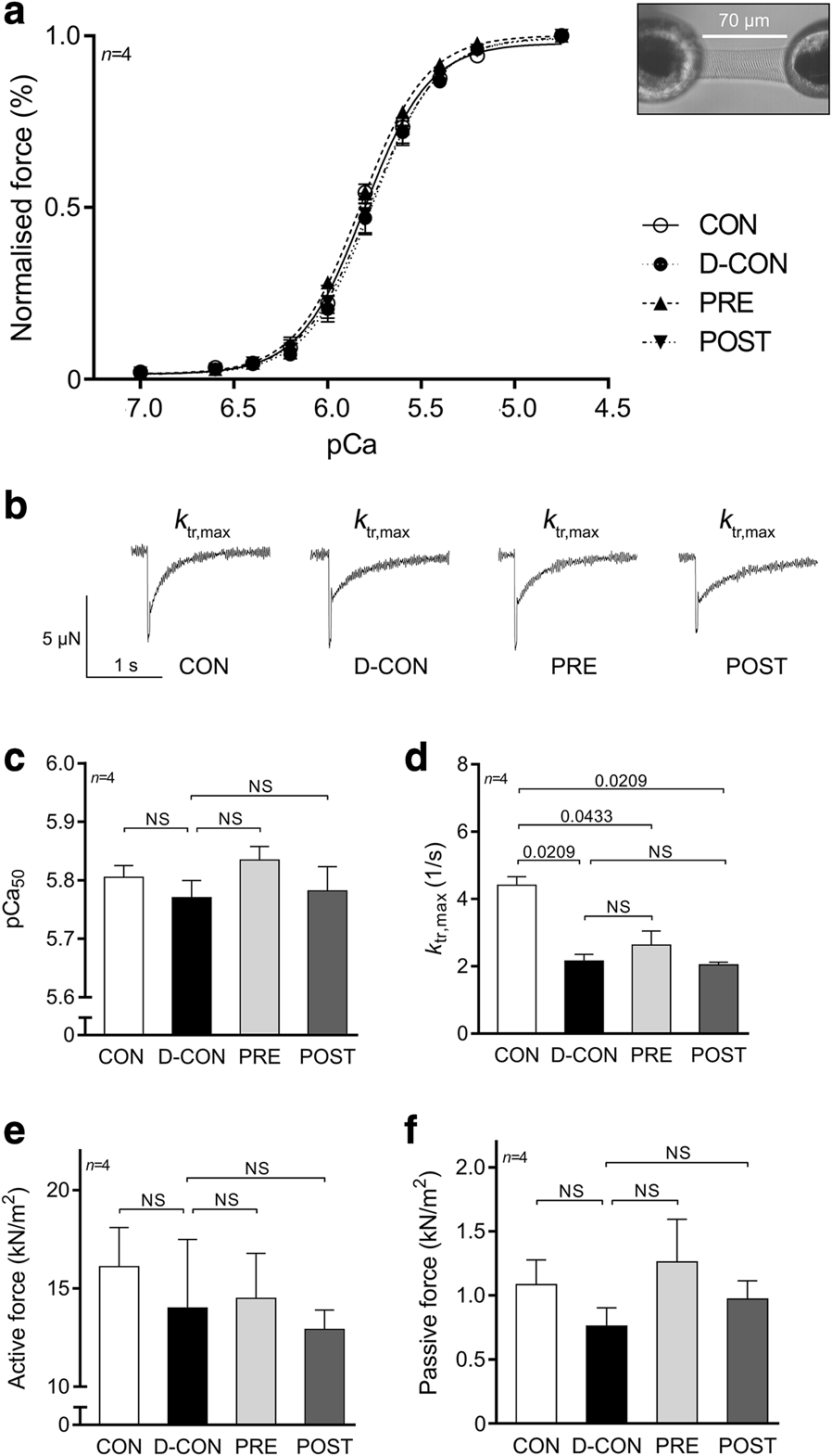
Force measurements in isolated cardiomyocytes. No significant change in the Ca^2+^-sensitivity (pCa_50_, **a**, **c**), active (**e**) or passive force values (**f**) could be detected, but a significant decrease in the *k*_tr,max_ was observed in all DOX-exposed groups, which could not be prevented by any treatment applied (**b**, **d**). Illustration of an isolated cardiomyocyte. pCa_50_ = Ca^2+^-sensitivity of isometric force production, *k*_tr,max_ = rate constant of force redevelopment. *n* number of animals per group (2–4 cardiomyocytes per animal); Statistics: Wilcoxon’s rank-sum test; numbers are p values; NS: non-significant

Mechanical measurements in single, permeabilised cardiomyocytes revealed no significant change in the Ca^2+^-sensitivity (pCa_50_), maximum Ca^2+^-activated active or Ca^2+^-independent passive force parameters of cellular preparations isolated from the groups (Fig. 6a, c, e–f; Table 3). However, a significant decrease in the *k*_tr,max_ was observed in all DOX-exposed groups compared to that in CON, which could not be prevented by any treatment applied (2.16 ± 0.20, 2.63 ± 0.42, 2.04 ± 0.08 vs. 4.41 ± 0.26 in D-CON, PRE, POST and CON, respectively, p ≤ 0.0433) (Fig. 6b, d).

**Table 3:** Contractile parameters of isolated cardiomyocytes

	CON (*n *= 4)	D-CON (*n *= 4)	PRE (*n *= 4)	POST (*n *= 4)
*F*_*max*_ (kN/m^2^)	16.1 ± 1.98	14 ± 3.47	14.49 ± 2.28	12.92 ± 0.98
*F*_*passive*_ (kN/m^2^)	1.08 ± 0.19	0.76 ± 0.14	1.26 ± 0.33	0.97 ± 0.14
*pCa*_*50*_	5.81 ± 0.02	5.77 ± 0.03	5.83 ± 0.02	5.78 ± 0.04
*k*_*tr,max*_ (1/s)	4.41 ± 0.26^+§^^	2.16 ± 0.2*	2.63 ± 0.42*	2.04 ± 0.08*
*nHill*	2.63 ± 0.21	2.56 ± 0.09	2.44 ± 0.14	2.47 ± 0.13

*F*_*max*_ maximum Ca^2+^-activated active force, *F*_*passive*_ Ca^2+^-independent passive force, *pCa*_*50*_ Ca^2+^-sensitivity of isometric-force production, *k*_*tr,max*_ rate constant of force redevelopment, *nHill* steepness of the force-pCa curve characterising the cooperativity between myofilament units, *n* number of animals per group (2–4 cardiomyocytes per animal)

* p ≤ 0.05 vs. CON; ^+^ p ≤ 0.05 vs. D-CON; ^§^ p ≤ 0.05 vs. PRE; ^^^ p ≤ 0.05 vs. POST

**Fig. 7:**
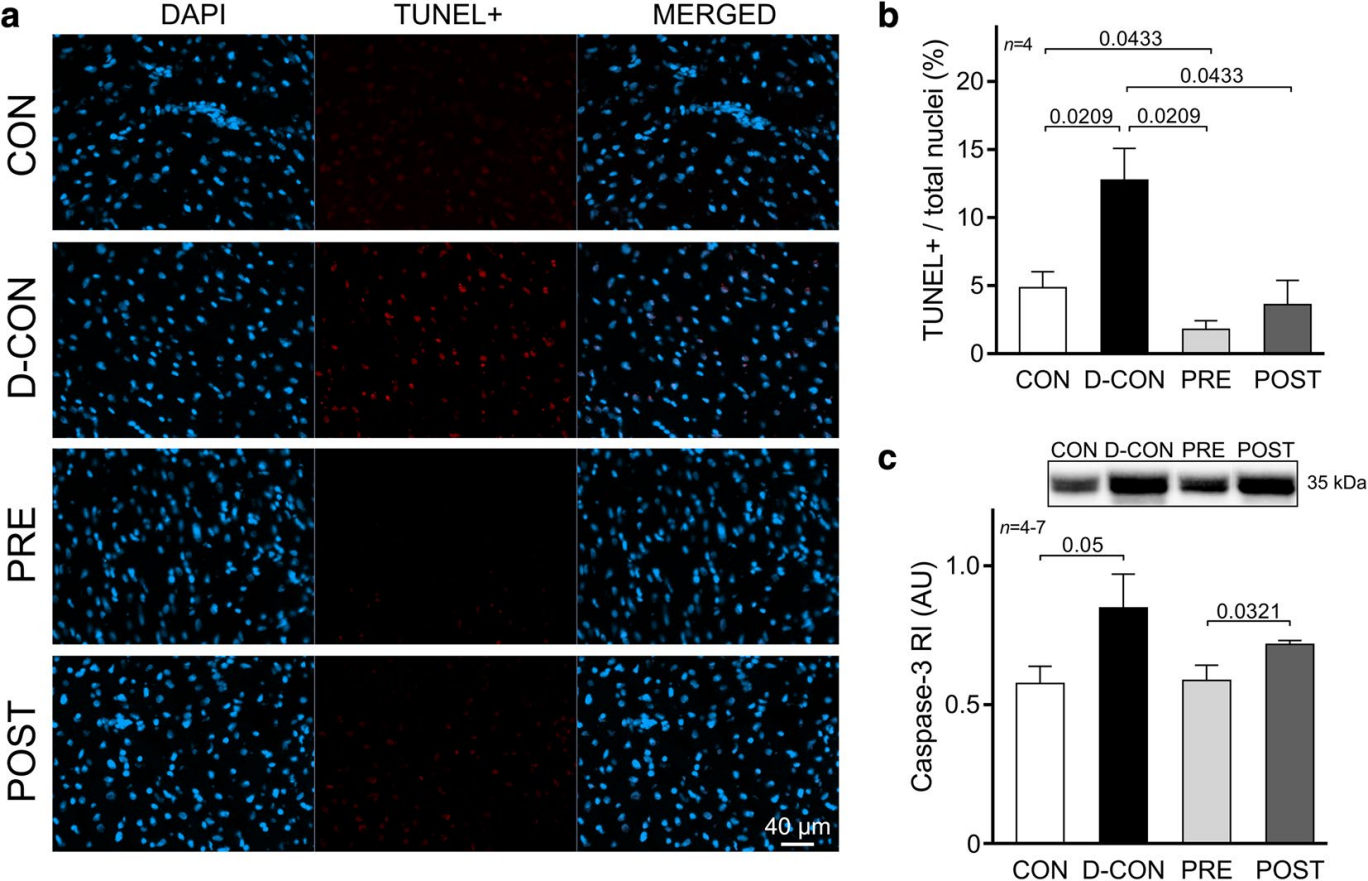
TUNEL assay and caspase-3 levels detecting apoptotic activity. **a** Representative images of cardiomyocyte apoptosis detected by TUNEL. The blue colour denotes all nuclei, the red colour denotes DNA damage, while the purple colour on the merged images denotes nuclei of apoptotic cardiomyocytes. **b** A significantly increased ratio of apoptotic nuclei in the D-CON group was detected, which was not apparent in the PRE or POST group. **c** The increased caspase-3 levels observed in the D-CON animals were markedly reduced in the PRE group, but not in the POST group. *AU* optical density in arbitrary units, *RI* relative intensity. *n* number of animals per group (8 images per animal for TUNEL, 3-5 measurements per animal for caspase-3); Statistics: Wilcoxon’s rank-sum test; numbers are p values

The TUNEL assay of the myocardial samples confirmed a significantly increased ratio of apoptotic nuclei in the D-CON group compared to that in CON (12.75 ± 2.35 vs. 4.85 ± 1.18%, p = 0.0209), which was not apparent in the PRE (1.79 ± 0.63%, p = 0.0209 vs. D-CON) or POST groups (3.62 ± 1.77%, p = 0.0433 vs. D-CON) (Fig. 7a, b). A Western blot analysis revealed an increased level of caspase-3 in the myocardial samples of the D-CON animals relative to that in CON [0.85 ± 0.12 vs. 0.58 ± 0.06 arbitrary units (AU), p = 0.05], which was markedly reduced by the prophylactic treatment in the PRE group (0.59 ± 0.05 AU, p = 0.089 vs. D-CON), but not by the conventionally scheduled therapy applied in the POST group (0.72 ± 0.01 AU, p > 0.9999 vs. D-CON, p = 0.0321 vs. PRE) (Fig. 7c).

**Fig. 8:**
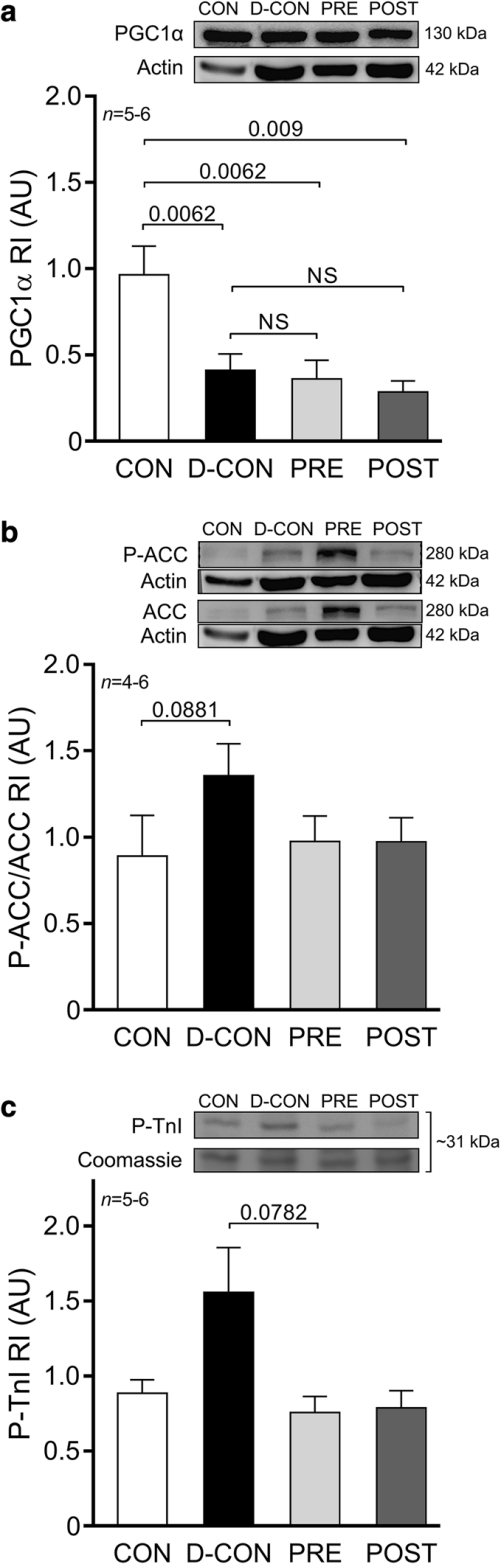
Biochemical measurements of proteins. A significantly decreased level of PGC1α was detected in the DOX-treated animals, irrespective of any treatment applied (**a**). A non-significant increase of P-ACC/ACC ratios was observed in the D-CON group compared to that in CON (**b**). A non-significant increase in the P-TnI was seen in the D-CON group compared to that in PRE (**c**). *ACC* acetyl coenzyme A carboxylase, *AU* optical density in arbitrary units, *P-ACC* phosphorylated ACC, *PGC1α* peroxisome proliferator-activated receptor-gamma coactivator 1 alpha, P-*TnI* phosphorylated troponin I, *RI* relative intensity. *n*  number of animals per group; Statistics: Wilcoxon’s rank-sum test; numbers are p values; NS: non-significant

To explore underlying molecular events behind the changes in the mitochondrial system of cardiomyocytes—which seem to be in the centre of DOX-induced cardiotoxicity—we characterised members of the cellular energy sensor web, PGC1α and adenosine monophosphate-activated protein kinase activity (by assessing the phosphorylation of ACC, its substrate). A Western blot analysis of PGC1α revealed significantly decreased levels in the DOX-treated animals, irrespective of any treatment applied (0.41 ± 0.1, 0.36 ± 0.11, 0.28 ± 0.07 vs. 0.96 ± 0.17 AU in D-CON, PRE, POST and CON, respectively, p ≤ 0.009) (Fig. 8a). Moreover, a non-significant increase of P-ACC/ACC ratios was observed in the D-CON group compared to that in CON (1.36 ± 0.18 vs. 0.89 ± 0.24 AU, p = 0.0881) (Fig. 8b). The ProQ™ Diamond gel staining revealed a non-significant increase in the phosphorylation level of a ~ 31 kDa protein co-migrating with cardiac troponin I in the D-CON group compared to that in PRE (1.56 ± 0.3 vs. 0.76 ± 0.11, p = 0.0782) (Fig. 8c). No changes were observed in the total phosphorylation of other contractile proteins. A gross analysis of the bands on the Oxyblot did not reveal any significant differences between the groups (data not shown).

## Discussion

The cardiotoxicity of modern oncotherapy represents not only a great clinical challenge, but also a heavy burden on cancer patients, their treating physicians and on society as well. Myocardial dysfunction and heart failure may be serious complications of chemotherapy with varying incidence [2]. Cardio-oncology is a relatively new and emerging discipline for the cardiovascular surveillance of oncological patients, in order to achieve successful oncotherapy with reduced myocardial harm, thereby improving the long-term survival rate and quality of life of patients. The present translational study sheds light on the importance of primarily applied prophylactic cardioprotection, which resulted in (1) an improved survival rate, (2) preserved systolic LV function and (3) conservation of the myocardial ultrastructure in our model. None of these beneficial effects were observed when the cardioprotective therapy was applied at a later stage. In the current work, efforts were made to design a clinically relevant study concept by closely mimicking a current oncotherapeutic approach of patients. An array of in vivo and in vitro methods were employed, including the relatively novel strain imaging, which is rarely applied in small animals mainly due to technical difficulties. We investigated several domains of DOX cardiotoxicity in vitro that may be interfered with the cardioprotective therapy applied (fibrosis, apoptosis, myofilament structure and function, oxidative stress, mitochondrial biogenesis).

Previously, Santos et al. investigated the structural and functional characteristics of mitochondria isolated from the hearts of DOX-receiving rats treated with carvedilol. According to their results, carvedilol was capable of preserving mitochondrial respiratory parameters and prevented mitochondrial damage. They also showed via electron microscopic imaging that the DOX-exposed myocardium displayed pronounced vacuolisation, which could not be seen in samples taken from the carvedilol group [54]. Similar beneficial effects on mitochondrial function and adenosine triphosphate production were shown by Hiona et al. when they tested the effects of enalapril in a rat model of DOX-induced cardiomyopathy [10]. In our model, we captured the above described electron microscopic pattern of vacuolisation in the D-CON group, but not in the PRE group. We also demonstrated a disturbed mitochondrial morphology in the D-CON group coinciding with reduced expression of a key nuclear orchestrator of mitochondrial biogenesis, PGC1α. These findings are consistent with previous data, where reduced expression of PGC1α accompanied cardiovascular lesions [55–57] and the induction of mitochondrial biogenesis was protective against DOX-cardiotoxicity [58]. Although the prophylactic treatment in our PRE group led to a preserved mitochondrial ultrastructure, it was not able to prevent the decrease in PGC1α.

Doxorubicin is thought to increase the production of reactive oxygen species [5]. These introduce oxidative stress and may damage various cellular structures, including the mitochondria. As mitochondria are essential in cellular energetics, their dysfunction also leads to free radical production and this further increases the level of oxidative stress. In a prior study the free radical production of DOX-exposed cultured rat cardiomyocytes could be alleviated by carvedilol but not by atenolol [59]. Similar beneficial effects of enalapril were described in vivo in rats [10], while captopril and telmisartan were found to be equally effective in reducing the level of oxidative stress markers [16]. In our study, we observed a slight increase in ACC phosphorylation in the D-CON group, which was normalised to some extent in the PRE and POST groups. This suggests that the pharmacological treatment applied may alleviate energetic stress presented to the cardiomyocytes. However, this effect was statistically not significant and a gross analysis of oxidative stress by protein carbonylation did not reveal any differences between the groups. When comparing our results with previous literature findings, the divergence in certain parameters may be accounted for by the differences in the models and protocols used, and also by possible unique features of the previously studied drugs, which may not always be apparent with other agents used in our study.

Myofibrillar degeneration and myofilament protein dysfunction are both representants of DOX-induced cardiotoxicity. A number of studies demonstrated detailed histological and electron microscopic data of DOX-induced myocardial changes, such as myofibrillar disarray [60], myofibrillolysis, sarcomeric disintegration [9, 61], contraction band formation [10], and cardiomyocyte hypertrophy [61, 62]. Some of these abnormalities were successfully attenuated by inhibiting the renin–angiotensin–aldosterone system [10, 15, 16]. In contrast with a conventionally scheduled therapy, we demonstrated a well-preserved ultrastructure of the myofilaments when applying a prophylactic treatment, which highlights the importance of the temporal aspects of cardioprotection in preserving myocardial ultrastructure.

In our study, we employed a unique mechanical measuring system to characterise contractile properties of isolated cardiomyocytes. Although we did not find any significant differences in most contractile parameters of the cardiomyocytes from the different groups, the significantly decreased *k*_tr,max_ value measured in the DOX-exposed cardiomyocytes suggests that the actin-myosin cross-bridge cycle of these myofilaments might be disrupted by DOX. Although a slight increase in the phosphorylation level of troponin I could be observed in the D-CON group, it did not reach statistical significance and was not accompanied with any robust change in the Ca^2+^-sensitivity of the cardiomyocytes isolated from this group.

Fibrosis represents another domain of DOX cardiomyopathy, which is also the subject of intensive preclinical research. Carvedilol [7], captopril [16], telmisartan [16] and spironolactone [63] have all been found to decrease DOX-induced myocardial fibrosis in small animals. Despite this, studies examining eplerenone gave either negative [12] or inconclusive results [13] when tested for the reversibility of DOX-induced interstitial fibrosis in mice. Interestingly, the combined application of our study drugs did not result in any significant improvement in the DOX-induced overall increase in fibrosis, regardless of the time span of the treatment. However, based on the mild differences seen in capillary density among the groups, we assume that the increase in the amount of myocardial collagen in the D-CON and POST animals might be the result of a more pronounced replacement fibrosis in response to cardiomyocyte cell death. In contrast, the relatively preserved capillarisation along with the increased collagen amount in the PRE group implies that the myocardial remodelling process in these animals could involve interstitial/perivascular fibrosis that might spare the cardiomyocytes. This hypothesis could also explain the preserved ejection fraction of these animals despite their increased fibrotic levels. However, the differences in capillary density among the groups were statistically non-significant, hence further investigations are necessary to draw firm conclusions from it. Overall, fibrosis in the present study is likely to play only a marginal role in the systolic LV dysfunction of the animals in the D-CON group, while other mechanisms, such as apoptosis could be more important here.

Apoptosis is often referenced as a main contributor to DOX-induced myocardial damage. Apoptotic cardiomyocytes can be identified by various assays (e.g. TUNEL) detecting DNA damage and fragmentation. Previously, many research groups examined the preventability of DNA damage in experimental DOX cardiomyopathy [7, 59, 63]. In accordance with their results, we successfully demonstrated that both the prophylactic and the conventionally scheduled therapy significantly reduce the degree of DNA fragmentation captured by TUNEL. At the same time, the caspase-3 activity, which is a biomarker of apoptosis, could only be substantially reduced by the prophylactic treatment, and not by the conventional therapy. Previously, Hiona et al. failed to demonstrate attenuating effects of enalapril on increased caspase-3 and caspase-9 activities in a rat model of DOX cardiomyopathy [10], while Spallarossa et al. found alleviating effects of carvedilol but not atenolol on caspase-3 activity in rat cardiomyocyte cultures exposed to DOX [59]. We think that in our model the previously described anti-apoptotic effect of perindopril [64] might play a key role in the attenuation of apoptosis of these cardiomyocytes, which could be attributed to the preservation of LV systolic function, especially in the PRE group, where animals received perindopril for an extended period of time.

## Conclusions

Overall, we demonstrated that a prophylactic, combined cardioprotective approach has many advantages compared to a conventionally scheduled heart failure therapy in attenuating DOX-induced cardiotoxicity. The significantly improved survival rate of the animals receiving prophylactic cardioprotection was not only accompanied by a preserved systolic LV function, but also by a conservation of the myocardial ultrastructure. We suggest that the marked reduction in cardiomyocyte apoptosis may be responsible for the observed beneficial effects of prophylactic cardioprotection. Our findings may indicate that a wider range of oncological patients awaiting anthracycline chemotherapy could benefit from prophylactic cardioprotection after an appropriate risk–benefit evaluation. Within the controversy of already available human data, our animal model might facilitate scientific progress in this field and it could be an important early step in the process of laying down the fundaments of new clinical practice. Still, no matter the intention for an adequate translational setting, preclinical testing in rodent models inevitably presents limitations to the extrapolation of data to the human condition. Thus, in the future, further validation by human clinical studies assessing prophylactic, low dose, combination heart failure therapy is necessary in oncological patients awaiting anthracycline chemotherapy.

## Additional file

**Additional file 1.** Online supplementary material.

## Data Availability

The datasets generated and analysed during the current study are available in the repository of the University of Debrecen. Access to the datasets is available from the corresponding author on reasonable request.
