# Supplementary material for: Advantages of prophylactic versus conventionally scheduled heart failure therapy in an experimental model of doxorubicin-induced cardiomyopathy
Source: J Transl Med. 2019 Jul 19;17:229. doi: 10.1186/s12967-019-1978-0 (PMC6642576; doi:10.1186/s12967-019-1978-0)
Supplement: Supplementary file 1 — Additional file 1. Online supplementary material. [file 12967_2019_1978_MOESM1_ESM.doc]

**Additional file 1**

**Advantages of prophylactic versus conventionally scheduled heart failure therapy in an experimental model of doxorubicin induced cardiomyopathy**

^1^Mária Lódi MSc, ^2^Dániel Priksz PharmD, ^1^Gábor Áron Fülöp MD, ^1^Beáta Bódi MSc, ^3^Alexandra Gyöngyösi MSc, ^8^Lilla Nagy PhD, ^1^Árpád Kovács MD, ^6^Attila Béla Kertész MD, PhD, ^4, 5^Judit Kocsis MD, PhD, ^6^István Édes MD, DSc, ^6^Zoltán Csanádi MD, DSc, ^6^István Czuriga MD, PhD ✞, ^7^Zoltán Kisvárday PhD, DSc, ^2^Béla Juhász PharmD, PhD, ^3^István Lekli PharmD, PhD, ^8^Péter Bai PhD, DSc, ^1^Attila Tóth PhD, DSc, ^1^Zoltán Papp MD, DSc, ^6^Dániel Czuriga MD, PhD

(1) Division of Clinical Physiology, Department of Cardiology, Faculty of Medicine, University of Debrecen, Debrecen, Hungary; (2) Department of Pharmacology and Pharmacotherapy, Faculty of Medicine, University of Debrecen, Debrecen, Hungary; (3) Department of Pharmacology, Faculty of Pharmacy, University of Debrecen, Debrecen, Hungary; (4) Department of 3^rd^ Internal Medicine, Semmelweis University, Budapest, Hungary; (5) Oncoradiology Center, Bács-Kiskun County Hospital, Kecskemét, Hungary; (6) Division of Cardiology, Department of Cardiology, Faculty of Medicine, University of Debrecen, Debrecen, Hungary; (7) Department of Anatomy, Histology and Embryology, Faculty of Medicine, University of Debrecen, Debrecen, Hungary; (8) MTA-DE Lendület Laboratory of Cellular Metabolism, Debrecen, Hungary

**1. Sample preparation and imaging for the TUNEL assay**

The cryostat sections were fixed in 4% cold buffered formalin, at 4°C for 45 min, then post-fixed in ethanol-acetic acid (2:1, v/v) at –20°C for 5 min. The sections were boiled in citrate buffer pH 6.0 for 12 min, then cooled at room temperature for 20 min. Samples were washed twice for 5 min in phosphate buffered saline (PBS) after each step. Next, sections were incubated with Tdt in a humidified box at 37°C for 1 hour. After washing, to identify nuclei, we used the DAPI (4',6-diamidino-2-phenylindole) dye (Sigma Aldrich, St. Louis, MO, USA), and Mowiol was used as a mounting medium. Images were captured by a Zeiss Axioskop microscope (Carl Zeiss Microscopy GmbH, München, Germany) using a software package (Carl Zeiss ZEN 2011). After merging the blue and red channels, purple spots were associated with apoptotic nuclei, while blue spots were identified as non-apoptotic nuclei. Apoptosis was quantified by the ratio of Tdt-positive nuclei / total nuclei in each section.

**2. Tissue processing for electron microscopy**

Tissue processing for electron microscopy was performed using a modified version of Somogyi’s technique [1]: a small piece from the left ventricular free wall was retrieved, then incubated in a cold phosphate buffered (PB; pH=7.4) fixative containing 2% paraformaldehyde (VWR International, Radnor, PA, USA) and 3% glutaraldehyde (Electron Microscopy Sciences, Hatfield, UK) overnight at 4°C. After washing three times in 0.1 M PB (pH=7.4), blocks were osmicated and then dehydrated in grading series of ethanol twice for 10 min followed by propylene-oxide. Samples were embedded into Durcupan^TM^ ACM (Sigma Aldrich, St. Louis, MO, USA) resin. From each block, 50 nm thick sections were cut and counterstained with uranyl-acetate and Reynold’s lead-citrate [2].

**3. Sample processing and signal detection for the Oxyblot measurements**

Protein carbonyl group investigations were adapted from Balogh et al. [3] using an Oxyblot Protein Oxydation Detection Kit (Merck Millipore, Burlington, MA, USA). Following the isolation of cardiomyocytes (similar to the force measurements), positive controls were treated with 1mM NaOCl and Fenton reaction mixture (containing 50 µM FeSO_4_, 6 mM ascorbic acid and 1.5 mM H_2_O_2_) at room temperature for 30 min. Incubated samples were then centrifuged (1000 G, 4°C, 1 min), and each sample was solubilised in sample buffer [8 M urea, 0.75 mM dithiothreitol (DTT), 4M thiourea, 3% sodium-dodecyl-sulphate (SDS), 10 µM E64, 40 µM leupeptin in 50 mM Tris-base, pH=14, 10% glycerol, 0.5% bromophenol blue]. After 1 hour of vortex, samples were centrifuged again (25°C, 5 min, 10,000 G). The protein concentration was determined from the supernatant with a dot-blot based method using bovine serum albumin (BSA) standards. Samples were diluted to a protein concentration of 3 mg/mL and derivatised using dinitrophenyl-hydrazine (DNPH) for 15 min at room temperature followed by neutralisation. Next, the samples were loaded onto 10% SDS-polyacrylamide gels, then transferred onto a nitrocellulose membrane. Blocking, primary and secondary antibodies were applied according to the protocol of the kit. Signals were then detected using the Westernbright ECL kit (Advansta, San Jose, CA, USA) on a gel documentation system (MF-ChemiBIS 3.2, DNR Bio-Imaging Systems, Jerusalem, Israel), and subsequently normalised for those assessed using the super-sensitive membrane staining kit (UD-GenoMed Medical Genomic Technologies Ltd., Debrecen, Hungary).

**4. Tissue preparation and signal detection for the ProQ^TM^ Diamond protein gel staining**

For the investigation of protein phosphorylation, ProQ^TM^ Diamond protein gel staining (Thermo Fisher Scientific, Waltham, MA, USA) was employed. Following the isolation of samples similar to that done in the Oxyblot measurements, they were diluted in 1X Laemmli buffer (Sigma Aldrich, St. Louis, MO, USA) and vortexed for 1 hour. The protein concentration was determined from the supernatant using a dot blot-based method with BSA as standard. The protein concentration was adjusted to 50 mg/mL and loaded onto 10% SDS-polyacrylamide gel. Gels were then labelled with ProQ^TM^ Diamond protein gel staining according to the protocol of the manufacturer. Bands were detected using a gel documentation system (MF-ChemiBIS 3.2, DNR Bio-Imaging Systems, Jerusalem, Israel) and normalised for Coomassie staining of the proteins. Protein identification was based on previous experiments of our laboratory [4].

**5. Sample preparation and signal detection for the Western immunoblot measurements quantifying mitochondrial proteins**

Samples from the left ventricular free wall were disrupted in an isolating solution, then sonicated for 10 seconds. After centrifugation (10,000 G, 10 min, room temperature), 20 µL 5X sample buffer (50% glycerine, 10% SDS, 310 mM Tris-HCl of pH=6.8, 100 mM DTT, 0.01% bromophenol blue) and 5 µL 2% β-mercaptoethanol were added to 80 µL supernatant and boiled for 10 min. The sample concentration was determined by a dot blot-based method using BSA as standard. 50 µg proteins were loaded onto 8% SDS-polyacrylamide gel and transferred onto nitrocellulose membrane. After blocking the membranes with 1% BSA in Tris-buffered saline containing 0.1% Tween 20 (TBST), the membranes were rinsed in the following antibodies overnight at 4°C diluted to 1:1000 in 1% BSA in TBST (all antibodies being produced in a rabbit): anti-acetyl coenzyme A carboxylase (ACC), anti-phospho-ACC and anti-peroxisome proliferator-activated receptor-gamma coactivator 1 alpha (PGC1α) (Cell Signaling Technology, Boston, MA, USA). After washing, the peroxidase-conjugated anti-rabbit IgG-specific antibody (Sigma-Aldrich, St. Louis, MO, USA; dilution: 1:40,000) was used at room temperature for 1 hour. The detection of the bands was performed in a similar way to the Oxyblot measurements. Afterwards, β-actin labelling was performed using a peroxidase conjugated antibody (Sigma Aldrich, St. Louis, MO, USA) for 1 hour at room temperature. Bands were normalised for β-actin labelling on the same membrane.

**6. Tissue preparation and visualisation for the Western immunoblot measurements quantifying caspase-3**

Approximately 100 mg of heart tissue was lysed in a 200 µl isolating buffer and the protein concentration was measured as previously described [5]. A total of 35 μg of protein in each sample was run on TGX Stain-Free^TM^ 12% acrylamide gels (Bio-Rad Laboratories, Hercules, CA, USA). Then, gels were exposed to UV light; hence trihalo compounds contained in stain-free gels covalently bound to tryptophan residues in proteins, which permitted total protein quantification. Transfer to a polyvinylidene difluoride (PVDF) membrane (Bio-Rad Laboratories, Hercules, CA, USA) lasted for 1 hour at 100V. After blocking the membranes with 5% of non-fat dry milk in Tris-buffered saline with 0.1% TBST, membranes were incubated overnight with the primary antibody solution (Caspase-3 1:500, Cell Signaling Technology, Boston, MA, USA). Afterwards, the membranes were washed with TBST three times and incubated with horseradish peroxidase (HRP)-conjugated secondary antibody solution (1:2000, Cell Signaling Technology, Boston, MA, USA) for 1.5 hours at room temperature. After washing, the membranes were incubated with Clarity Western ECL Substrate (Bio-Rad Laboratories, Hercules, CA, USA) to visualise them by enhanced chemiluminescent bands according to the recommended procedure (ChemiDoc Touch, Bio-Rad Laboratories, Hercules, CA, USA). The chemiluminescent bands were normalised to the total protein in each lane with Image Lab^TM^ 5.2.1. Software (Bio-Rad Laboratories, Hercules, CA, USA) [6]. The relative intensity was then compared to an internal control.

**7. References**

1. Somogyi P The study of Golgi stained cells and of experimental degeneration under the electron microscope: a direct method for the identification in the visual cortex of three successive links in a neuron chain. Neuroscience. 1978;3:167-80.

2. Reynolds ES The use of lead citrate at high pH as an electron-opaque stain in electron microscopy. J Cell Biol. 1963;17:208-12.

3. Balogh A, Santer D, Pasztor ET, Toth A, Czuriga D, Podesser BK, et al. Myofilament protein carbonylation contributes to the contractile dysfunction in the infarcted LV region of mouse hearts. Cardiovasc Res. 2014;101:108-19.

4. Kovacs A, Kalasz J, Pasztor ET, Toth A, Papp Z, Dhalla NS, et al. Myosin heavy chain and cardiac troponin T damage is associated with impaired myofibrillar ATPase activity contributing to sarcomeric dysfunction in Ca(2+)-paradox rat hearts. Mol Cell Biochem. 2017;430:57-68.

5. Czompa A, Gyongyosi A, Szoke K, Bak I, Csepanyi E, Haines DD, et al. Effects of Momordica charantia (Bitter Melon) on Ischemic Diabetic Myocardium. Molecules. 2017;22.

6. Gurtler A, Kunz N, Gomolka M, Hornhardt S, Friedl AA, McDonald K, et al. Stain-Free technology as a normalization tool in Western blot analysis. Anal Biochem. 2013;433:105-11.
